# Machine Learning Readmission Risk Modeling: A Pediatric Case Study

**DOI:** 10.1155/2019/8532892

**Published:** 2019-04-15

**Authors:** Patricio Wolff, Manuel Graña, Sebastián A. Ríos, Maria Begoña Yarza

**Affiliations:** ^1^Research Center on Business Intelligence, University of Chile, Beauchef 851, Of. 502, Santiago, Chile; ^2^Hospital Dr. Exequiel González Cortés, Gran Avenida 3300, San Miguel, Santiago, Chile; ^3^Computation Intelligence Group, Basque University (UPV/EHU) P. Manuel Lardizabal 1, 20018 San Sebastian, Spain; ^4^ACPySS, San Sebastián, Spain

## Abstract

**Background:**

Hospital readmission prediction in pediatric hospitals has received little attention. Studies have focused on the readmission frequency analysis stratified by disease and demographic/geographic characteristics but there are no predictive modeling approaches, which may be useful to identify preventable readmissions that constitute a major portion of the cost attributed to readmissions.

**Objective:**

To assess the all-cause readmission predictive performance achieved by machine learning techniques in the emergency department of a pediatric hospital in Santiago, Chile.

**Materials:**

An all-cause admissions dataset has been collected along six consecutive years in a pediatric hospital in Santiago, Chile. The variables collected are the same used for the determination of the child's treatment administrative cost.

**Methods:**

Retrospective predictive analysis of 30-day readmission was formulated as a binary classification problem. We report classification results achieved with various model building approaches after data curation and preprocessing for correction of class imbalance. We compute repeated cross-validation (RCV) with decreasing number of folders to assess performance and sensitivity to effect of imbalance in the test set and training set size.

**Results:**

Increase in recall due to SMOTE class imbalance correction is large and statistically significant. The Naive Bayes (NB) approach achieves the best AUC (0.65); however the shallow multilayer perceptron has the best PPV and f-score (5.6 and 10.2, resp.). The NB and support vector machines (SVM) give comparable results if we consider AUC, PPV, and f-score ranking for all RCV experiments. High recall of deep multilayer perceptron is due to high false positive ratio. There is no detectable effect of the number of folds in the RCV on the predictive performance of the algorithms.

**Conclusions:**

We recommend the use of Naive Bayes (NB) with Gaussian distribution model as the most robust modeling approach for pediatric readmission prediction, achieving the best results across all training dataset sizes. The results show that the approach could be applied to detect preventable readmissions.

## 1. Introduction

Hospital readmission is defined as the nonscheduled return of a patient within a short prespecified period of time after hospital discharge. An internationally extended standard period to count a patient return as readmission is 30 days, but it may change for political reasons [1]. In the United States (US), hospital readmission is being used as an indicator of patient care quality. Both public and private funding agencies use this measure to penalize underperforming institutions [2]. It has been argued that up to two-thirds of the readmissions are preventable; therefore advances in patient readmission prediction are worth the investment [3, 4]. US policy has inspired similar concerns in other countries so that readmission analysis and prediction is under consideration worldwide. The data collected in the Electronic Health Record (EHR) is the main information source for the predictive modeling of readmissions and the analysis of their consequences and structural/organizational causes [3, 5].

Readmission prediction in the case of adult patients has been tackled with diverse statistical approaches [1, 6] such as logistic regression [7, 8] and survival analysis [9]. Recent works favor the application of predictive machine learning approaches, formulating readmission prediction as a binary classification problem [7, 10]. For example, the literature report results from support vector machines (SVM) [4, 11, 12], deep learning [13, 14], artificial neural network [8], and Naive Bayes [5, 15].

Despite this long history of studies about hospital readmission for adult patients, there are almost no studies devoted to readmission of pediatric patients [2]. In the pediatric case, hospital readmission prediction has been only reported in the setting of emergency department [16, 17] and intensive care units [18]. Few studies report results on both adult and pediatric patients [7], finding lower sensitivity in the pediatric population than in the adult population, due to greater class imbalance in the pediatric datasets. In this paper we report the predictive modeling results over a large cohort of all-cause admissions to the emergency department of a pediatric hospital in Santiago, Chile. We tested four modeling applications considering various numbers of folds in a repeated cross-validation approach, achieving results comparable to those reported for adult patient readmissions.

## 2. Materials and Methods

**Figure 1:**
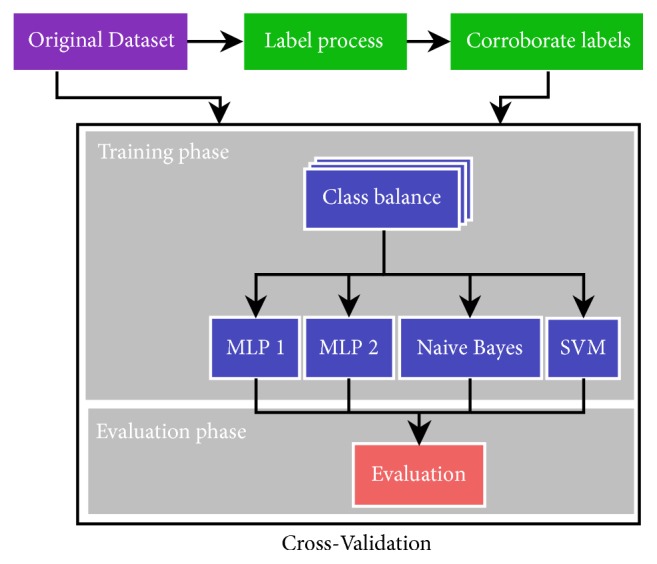
Study design.

The overall model training and validation process is shown in Figure 1. First, the EHR data entries were labeled as readmissions according to the following rules: (a) we consider admissions in period of less than 30 days after the previous discharge; (b) we discard an admission if it corresponds to programmed treatments such as chemotherapy, or if it is intended for services that are not urgent. We check (corroborate) the correctness of the generated labels by an expert committee, which consisted of two experienced medical doctors and two nurses from the hospital's quality and safety care team. The whole data is then used for validation in a repeated cross-validation (RCV) process with different numbers of folders; we carried out 10-fold, 5-fold, 4-fold, and 3-fold RCV. Each cross-validation repetition consists in the following steps: (1) partition of the dataset in the selected number of folds, (2) each fold is alternatively used as the test dataset while the remaining folders are used for model training, and (3) average performance measures are computed over all cross–validation folds and repetitions. As illustrated in Figure 1, training at each RCV step is preceded by a class balance process carried out on the training dataset. We apply a SMOTE [19] upsampling procedure using the five nearest neighbors of each minority class sample [7, 10]. The reported results are the average of the 30 repetitions of the CV results. We have published the script of the implementation as open source code for independent examination [20].

### 2.1. Cohort and Dataset

The descriptive statistics of the dataset used for the study are summarized in Table 1. It contains records of 56,558 admissions with 2106 readmissions in the period from July 2011 to October 2017 at the pediatric Hospital Dr. Exequiel González Cortés in Santiago, Chile. All data has been anonymized for the study. One author (PW) acts as the honest data broker ensuring compliance with data protection regulations. The categories of data available to build machine learning based predictors are the following ones:

- Data used by the administrative cost coding system, specifically, age, sex, ethnic group, anonymized geographical information (i.e., postal code), public insurance plan, principal diagnosis, secondary diagnosis, tertiary diagnosis, and main procedure performed.
- Information about patient's admission: the date of admission, the service in which he/she was admitted, and his/her origin.
- Information on internal transfers: date/hour, service of origin and internal destination.
- Information about the patient's discharge: discharge date, service that performs the discharge, and the patient's destination.

Though we have not carried out a detailed statistical survey of the occurrence of readmissions according to specific diagnostics [21], we have been able to identify the diagnostic at discharge accounting for most of readmissions as detailed in Table 2. There is a big prevalence of respiratory conditions that can be attributed to pollution events in the city of Santiago.

To improve data quality a manual data curation process was carried out. Identification of admissions that are actual readmissions was carried out automatically. The resulting labeled dataset is heavily class imbalanced. A taxonomy of methods to deal with imbalanced data presented in the context of readmission prediction is given in [6]. For training, we applied a class balancing technique, specifically a SMOTE [19] on the minority class using five nearest neighbors. We have considered increasing sizes of the balanced training set, leaving the remaining (imbalanced) as the test set.

### 2.2. Classification Methods

Several machine learning [22, 23] approaches have been selected for predictive model building. These models have been reported in the literature about readmission prediction for adult patients [1, 6]. We have discarded application of deep learning approaches [24] because the available data is too shallow. There is no spatial information, the time sequences of readmissions are too short to be exploitable, and the number of variables per patient data entry is too small to generate high dimensional hierarchical representations. Therefore we focus on well-known classical methods. The reported applications of deep learning to readmission prediction are restricted to a specific disease, i.e., lupus patients [13], for which there are long clinical histories* per* patient accessible through the EHR, so that the abundance of data allows for the training of deep models.

#### 2.2.1. Support Vector Machines [25]

Support Vector Machines (SVM) classifiers are linear discriminant functions built from samples placed at the boundaries of the classes. Their learning algorithm looks for the discriminating hyperplane maximizing its distance to the boundaries belonging to each class, i.e., maximizing the margin of the decision function relative to the class boundary. The parameters that define the solution hyperplane come from the optimization of a quadratic programming problem. When the classes are not linearly separable, then it is possible to project the data into a space of superior dimensionality using the kernel trick [26], so that the transformed dataset becomes linearly separable. The literature shows that SVMs are quite robust against the curse of dimensionality, achieving good results on small datasets of high dimensionality feature vectors. We used LibSVM [27] library for training and estimation of the SVM metaparameters via grid search. Best results were obtained with a Radial Basis Function (RBF) kernel. We have used LibSVM (https://www.csie.ntu.edu.tw/~cjlin/libsvm/) for SVM training.

#### 2.2.2. Multilayer Perceptron

Multilayer perceptron (MLP) is the classical feed-forward artificial neural networks (ANN) composed of multiple densely interconnected layers of computational units, aka artificial neurons. The output of each unit is computed as the linear combination of the incoming connection weights and their source units in the previous layer filtered by a nonlinear activation function. The classical sigmoid activation function has been replaced by others like the rectified linear activation used in deep learning architectures. The connection weights implement a discriminant function that may take arbitrary shapes. In fact it has been shown that even with a single hidden layer, an MLP can approximate any function. The connection weights can be learned from data applying the back-propagation algorithm [23].

We have applied two flavors of MLP to pediatric readmission prediction. The first one (denoted MLP1 in the results section) is an autotunable implementation, called AutoMLP for short, which performs automatic online model parameter tuning during training process, including the creation of an ensemble of MLPs [28]. The number of maximum training cycles used for the ANN training was 10 equals to the number of generations for AutoMLP training and the number of MLPs per ensemble chosen was 4.

The second (denoted MLP2 in the results section) is a multilayer feed-forward artificial neural network trained using back-propagation with stochastic gradient descent [24]. The activation function used by the neurons in the hidden layers was a Rectifier function. The MLP2 has two hidden layer, each of 50 neurons. It was trained in 10 epochs using an adaptive learning rate algorithm (ADADELTA) [29] which combine the benefits of learning rate annealing and momentum training to avoid slow convergence. We used the *H*_2_0 package (https://www.h2o.ai) for this MLP training and validation [30].

#### 2.2.3. Naïve Bayes Method

The Naïve Bayes (NB) approach is based on the assumption that the individual features are statistically independent; therefore we approximate the joint probability distribution of a high-dimensional feature vector as the product of the unidimensional distribution probabilities of each feature. In our study we use unidimensional Gaussian probability density models of the independent feature distributions. Training was carried out by straightforward estimation of these unidimensional probability densities.

### 2.3. Classification Performance Metrics

At each cross-validation fold we compute the confusion matrix and performance metrics derived from it, finally reporting the average of these results. Let us define TP, TN, FP, and FP as true positive, true negative, false positive, and false negative counts. Then we compute the Recall (aka sensitivity) as

(1) $$\begin{array}{c} R=\frac{TP}{TP+FN}, \end{array}$$

positive predictive value as

(2) $$\begin{array}{c} PPV=\frac{TP}{TP+FP}, \end{array}$$

and f-score as

(3) $$\begin{array}{c} F=\frac{2}{1/R+1/PPV} \end{array}$$

These measures are more informative than the accuracy (*A* = (*TP* + *TN*)/(*TP* + *TN* + *FP* + *FN*)) of the successful detection of the minority class (i.e., the readmissions) because the dataset is strongly class imbalanced. The analysis using Receiver Operating Characteristic (ROC) curves has been widely used to compare different binary classifiers. The ROC is a plot of sensitivity versus the false positive rate (*FPR* = *FP*/(*FP* + *TN*)). It is widely used to compare performances of state of art of supervised learning classification methods. Specifically the integral of the ROC, i.e., the Area Under ROC Curve (AUC), is often reported in readmission prediction studies of adult patients [6].

We compute these measures over the test dataset after training the models in an RCV process explained above. At each fold test, the remaining folds are put together as the training dataset. The training dataset is class-balanced using SMOTE [19] with five nearest neighbors on the minority class training samples until we have the same number of samples of each class. However, the test set remains unaffected and heavily imbalanced. One consequence is that small errors in absolute terms (e.g., one misclassified sample) translate into large reductions of the performance measures. The proportion of samples of the minority class in the test dataset depends on the number of folds used for RCV. High number of folds implies big reductions in the number of minority class samples in the test fold, thus increasing its imbalance ratio (the ratio of the majority class sample size to the minority class sample size), which may lead to numerical instabilities of the performance results. For this reason, we have explored the results obtained using a decreasing number of RCV folds.

## 3. Results

Tables 3, 4, 5, and 6 show the average recall, positive predictive value, f-score, and AUC, respectively, of the machine learning techniques after 30 repetitions of the RCV experiments with varying number of folders with and without SMOTE class imbalance correction. The effect of the number of folds is negligible. An F- test over the number of folds shows that there is no statistically significant difference (p>0.1).

The difference between results due to the use of SMOTE class imbalance correction at model building is largely statistically significant (p<0.00001 one sided t-test of PPV, f-score, and AUC values almost for all models). For the results without SMOTE are somehow paradoxical. The PPV grows significatively in some cases (for SVM >40%), but the recall is extremely low (for SVM <2%). The interpretation is that the number of cases classified as positive is very small, so that a small number of true positives gives high PPV. For MLP1 we found many instances of NA values due to the lack of positive responses.

**Figure 2:**
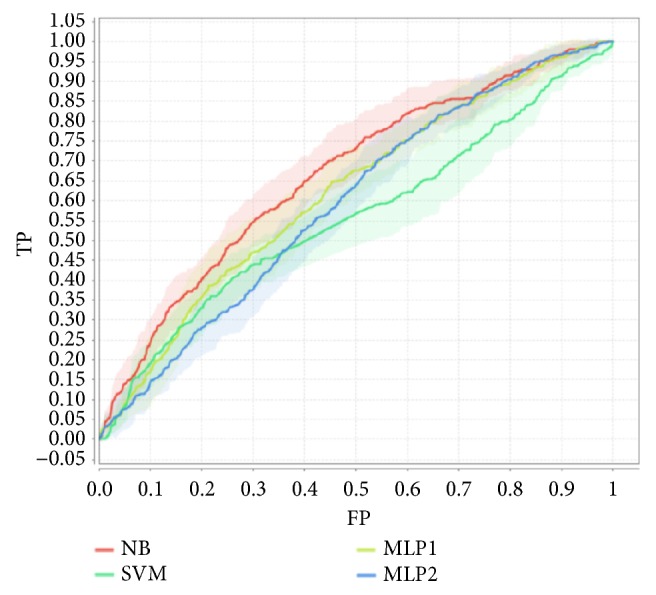
Average ROCs of machine learning approaches in 5-fold RCV (applying SMOTE class imbalance correction). Solid line corresponds to the ROC mean.

Let us consider the case when we apply the SMOTE class imbalance correction. Attending to recall (R) in Table 3, MLP2 is well above SVM, MLP1, and NB; however, this is at the cost of a high false positive ratio, as demonstrated by the values of the PPV in Table 3, which is much lower for MLP2 than for SVM, MLP1, and NB.  Figure 2 shows the ROC curves for all approaches in the case of RCV with 5 folders.

The f-scores shown in Table 3 confirm that SVM, MLP1, and NB improve over MLP2 regardless of RCV number of folders. An F-test carried out over these results confirms (p<0.01) that the performance differences between predictive models are statistically significant. Ensuing specific one-sided t-tests comparing each pair of modeling approaches confirms that SVM, MLP1, and NB perform significantly better than MLP2. The AUC results in Table 3 confirm that NB is significantly better than the remaining approaches (F-test p<0.01, pairwise t-test p <0.001). However, the superiority of NB relative to MLP1 is less pronounced (pairwise t-test p<0.05). Notice that statistical significance is due also to small standard deviation of the results; if we consider the mean performance values, we can assert that SVM and NB show comparable performances.

## 4. Discussion

### 4.1. Readmission as a Healthcare Quality Measure

Readmissions as a healthcare quality measure have been the subject of strong debate both in adult and in pediatric hospital environments [2]. The cost of readmissions within a 365 day period is estimated as $1 billion in United States pediatric hospitals [31], hence the need for focused analysis and predictive tools. There are, however, some studies that question the value of readmissions as a quality of care metric for specific type of patients, e.g., those suffering heart failure [32]. Other studies argue that too much emphasis in readmissions as a measure of the quality of care may lead to an increase of the unequal distribution of resources [1]. There is a need to be precise in the definition of which readmissions are to be penalized. For instance, if there is not distinction between planned and unplanned readmissions, there is a possibility that the hospitals would tend to delay required readmissions after the 30-day limit to avoid financial penalties [33]. It is also well known fact that a small percentage of pediatric patients with chronic conditions and special technological assistance needs account for a big percentage of the actual readmission costs [34]. The emphasis is, therefore, in the identification of the kind of readmission events that can be prevented through special care after discharge, such as phone calls [35].

### 4.2. Quantitative Analysis of Readmissions in Pediatric Care

Thought readmission prediction has been extensively studied in adult patients, there is very little effort in children hospitals. One reason is that the percentage of admissions that result in readmission is much less frequent event in the pediatric case, in the range 3% to 5% on average, than in adult patients, which is close to 17% on average [4], so it was dismissed in cost analysis studies until recently. To our knowledge, our study is among the first ones applying machine learning techniques to all-cause pediatric readmissions. We have only found one similar study with a smaller cohort [17] in an Italian hospital. Recent studies are devoted to the characterization of the readmission events in the pediatric setting. Auger et al. [33] propose a method for the identification of unplanned versus planned readmissions which has many implications in the way readmissions are treated in order to avoid financial penalties. For instance, planned readmissions may be delayed to avert financial penalties. It is also important to identify which pediatric conditions lead to higher readmission rates, realizing that they may be changing from one institution to another due to local demographic and environmental conditions; for instance, some studies found strong dependence of frequency of readmissions on the ethnic, disease, chronic condition, and other demographic information such as the public versus private insurance [34, 36, 37]. Dependency of readmission frequency on clinical and geographic factors for a specific chronic condition (i.e., sickle cells disease) has been reported [38]. On the other hand, shorter length of stay in pediatric hospitals is not a cause for higher readmission rate [21]. Another issue is the impact of the use by the administrations in charge of financial control of the hospital of proprietary algorithms for the detection of preventable readmission detection. Being proprietary, the actual reasoning behind the decision is unknown, and thus it is quite difficult to predict its outcome in order to optimize patient care and financial management simultaneously [39].

The difficulties are faced when trying to look for agreement among readmission prediction research studies or assessing the significance of a new study as follows:

1. The conditions for readmission are local to the population treated by the hospital. It is unrealistic to apply the same risk assessment/prediction model in two countries with huge differences in life parameters and conditions. Therefore, it is widely recognized that predictive models need to be developed at each site using local data [1, 16].
2. Because hospital readmission is a much less frequent event than no readmission, data used in all reported studies is heavy class imbalance [17]. In our study, the readmissions account for only 3,7% of the samples. Therefore, class balancing techniques are required to avoid model bias towards the majority class [40].
3. Often, EHR data has a lot of errors and missing information due to the stressful conditions of its capture. Moreover, there is no guarantee that the collected variables are indeed the most relevant for the intended prediction. However, it is the only available data for this purpose most of the times. Recent reviews and comparative studies [1, 4, 6] have found that studies on adult readmissions reported low values of area under ROC Curve (AUC aka c-statistic) ranging between 0.56 and 0.72. One way to improve prediction results is to carry out stratified studies, i.e., building specific predictive models for specific patient categories [41].

### 4.3. Class Imbalance

The readmission rate in our case study is 3,7% which is similar to the percentage of readmissions reported in other studies about pediatric readmissions, i.e., 2.6% in [37]. Class imbalance poses great difficulties both during training and validation. At training time, machine learning approaches are biased towards the majority class, so data preprocessing is required to create balanced training datasets [6, 7]. We choose to upsample the minority class using SMOTE [19]. Additionally, care must be taken in the selection of the performance metric. Overall accuracy is strongly influenced by the majority class correct classification; therefore we need to use performance measures that take into account the performance regarding the minority class; hence we consider the positive predictive value (PPV), f-score (F), and the area under the ROC (AUC). The cost of false positive decision is much lower than false negatives; therefore we have not considered setting a false positive ratio for all algoriths. The AUC measure has been reported in most predictive studies of readmission. Our top result (AUC=0.655 for NB) is similar to the results already reported for adult readmissions (between 0.56 and 0.72). For a dramatic illustration of the effect of the class imbalance, we report the results without using SMOTE class imbalance correction. We find a huge decrease in recall performance, meaning that the readmission prediction drops drastically relative to the models built upon SMOTE corrected training data, beause of large bias towards the majority class in the non-SMOTE models. The small number of positive predictions leads to some paradoxical results, such as the increase of PPV value relative to the SMOTE models, because the false positive predictions are also very scarce.

### 4.4. Limitations of the Study

The dataset comes from a single hospital, so results reported need to be assessed with data coming from a network of hospitals in the same country, including data from other countries risk the introduction of uncontrollable variations due to diverse data gathering protocols and differences in prevalent morbid conditions. For instance, sickle cell crisis is a costly and frequent readmission condition in USA [39] while it is nonexistent in Chile. Therefore, it is quite necessary to carry out local studies in order to assess predictability and preventability instead of importing models from other countries which may be misleading. The existence of EHR data collection, anonymization, and distribution infrastructures in United States, such as the Pediatric Health Information System of the Children's Hospital Association (https://childrenshospitals.org) or the Nationwide Readmissions Database (https://www.hcup-us.ahrq.gov/nrdoverview.jsp), has favored the realization of studies covering many institutions and large cohorts [21, 31, 34, 36, 37, 39]. We hope that the study in this paper will encourage the creation of similar infrastructures outside United States.

### 4.5. On the Practical Implementation of the Predictive System

Reviewers have raised the relevant question of the cost-benefit tradeoff of the implementation of the predictive approach in the clinical practice. In their words, a relevant question is whether it is worth intervening almost twenty patients in order to reduce the likelihood of one readmission (according to PPV values). From the technical point of view, the system would be implemented as an assistive device, so that the intervention decision is always in the clinician hands. Clinicians have expressed the desire to have some kind of objective reference to help them focus on the risky cases. On the other hand, implementation of a predictive system as described in the paper would give a dichotomy decision. However, there is a gradation of risk underlying this decision, which may be modeled by the* a posteriori* probability estimations computed by the predictive models. In fact, the dychotomic decision is the result of the application of an arbitrary threshold (often 0.5) to these* a posteriori *probability estimations. Future work should be addressing the task of providing a risk gradation to the clinicians, easing the task of targeting really critical cases that need more specific intervention, such as giving detailed training to the parents for child treatment at home, or delaying the child discharge from the hospital. From the administrative point of view, the hospital is increasing the decision assistant tools provided to the clinicians. For instance, there is a tool providing triage recommendations. Therefore, they are definitively in favor of the implementation of the kind of tools described in the paper. Furthermore, the continuous inflow of information and the addition of new variables will allow the improved tuning of the tool. Finally, from the human point of view, any parent will be in favor of the implementation of such tools if they improve somehow the health care quality of their children.

## 5. Conclusions

Following the track of political decisions in United States regarding cost effective quality healthcare, hospital readmissions have become a concern worldwide. There have been many quantitative analysis, mostly for adult patients, including predictive approaches based on machine learning. However, pediatric hospital readmissions have received little attention until recently. One of the lessons learned is that there is much variability between locations so that it is preferable to develop local predictive models than trying to apply models developed upon foreign country data. Another lesson learned is that it is desirable to have research oriented nationwide data collection and distribution resources that may allow carrying out precise and extensive quantitative analysis.

In this paper, we report the results of an all-cause predictive modeling study carried out over the anonymized dataset collected over six years of operation in a public pediatric hospital in Santiago, Chile. The amount of data gathered is large for a single site study (56,558 discharges and 2,106 readmissions), but it would be desirable to enlarge it with the contribution of other institutions in Chile. We have applied four predictive methods upon the administrative data used for patient cost estimation. The results are good, achieving a top predictive performance AUC=0.65 that is comparable to other predictive studies on adult patients data. However, this is the result of a dychotomic decision, which puts together mild risk cases with high risk cases. Future work should be addressed to give a more precise quantification of the risk of readmission, allowing for focus on more efforts on the riskiest cases.

To our knowledge this is the first study in Chile of this kind and among the first ones worldwide, devoted to pediatric readmissions. In the future, it will be desirable to have access to a nationwide data repository, in order to be able to derive general models upon which specific policies for optimal cost management maintaining while improving the service quality could be formulated. The inclusion of other data modalities, such as medication, international disease code, laboratory, and clinical data, would help to extend this study into the so-called phenomics realm, which aims to exploit the big data contained in the EHRs in order to achieve personalized medical recommendations and follow-up. Such large data collections would allow also the application of recent breakthrough technologies such as deep learning.

## Figures and Tables

**Table 1:** Descriptive statistics of the dataset.

Dataset characteristic	
Total number of admissions	56,558
Number of unique individuals	35,064
Percent readmission within 30 days	3.72%
Number of unique procedures (ICD-10 AM)	1,124
Number of unique diagnoses (ICD-10 AM)	4,370

Variables used in prediction	

Age (years), mean (SD)	5.78 (5.04)
Male (%)	59.2
Public facilities	1
Number of Transfers (SD)	0.61 (0.8)
Length of Stay (days), mean (SD)	3.77 (10.03)

**Table 2:** Diagnostics at discharge accounting for most readmission.

Diagnostic	ICD10	%
Viral pneumonia	J129	9.50
Respiratory syncytial virus pneumonia	J121	9.16
Acute bronchitis	J209	3.94
Unspecified gastroenteritis	A090	2.80
Disorders of prepuce	N47	0.90

**Table 3:** Average ± standard deviation Recall (R) performance [%] of SVM, MLP1, MLP2, and NB for decreasing number of folders in the RCV process. no SMOTE = no oversampling correction of class imbalance is done.

nfolds	SMOTE			
	SVM	MLP2	MLP1	NB
10	45.63 ±3.35	96.29 ±2.15	59.93 ±5.51	70.8 ±2.68
5	44.64 ±2.69	96.58 ±1.77	61.39 ±6.14	69.8 ±4.97
4	43.83 ±1	95.11 ±1.06	59.87 ±6.29	70.23 ±3.82
3	43.64 ±1.11	96.86 ±0.37	52.8 ±5.24	67.57 ±0.97

	no SMOTE			
	SVM	MLP2	MLP1	NB

10	0.95 ±0.76	27.60 ±11.13	0.00 ±0.00	14.81 ±1.83
5	1.04 ±0.71	33.24 ±8.65	0.00 ±0.00	14.77 ±1.43
4	1.00 ±0.21	29.11 ±13.90	0.00 ±0.00	14.91 ±1.6
3	1.14 ±0.23	30.32 ±17.48	0.00 ±0.00	14.67 ±1.89

**Table 4:** Average ± standard deviation positive predictive value (PPV) [%] of SVM, MLP1, MLP2, and NB for decreasing number of folders in the RCV process. no SMOTE = no oversampling correction of class imbalance is done.

nfolds	SMOTE			
	SVM	MLP2	MLP1	NB
10	5.52 ±0.35	3.92 ±0.09	5.61 ±0.47	5.28 ±0.16
5	5.43 ±0.27	3.98 ±0.1	5.25 ±0.14	5.29 ±0.31
4	5.39 ±0.1	3.99 ±0.01	5.29 ±0.19	5.29 ±0.07
3	5.48 ±0.1	3.94 ±0.03	5.34 ±0.07	5.4 ±0.09

	no SMOTE			
	SVM	MLP2	MLP1	NB

10	42.22 ±29.86	6.23 ±1.53	NA	9.05 ±1.11
5	32.47 ±16.63	5.40 ±0.59	0.00	9.02 ±0.95
4	45.24 ±5.35	6.60 ±1.96	0.00	9.09 ±1.13
3	45.24 ±12.14	6.22 ±0.82	NA	8.90 ±0.89

**Table 5:** Average ± standard deviation f-score (F) performance [%] of SVM, MLP1, MLP2, and NB for decreasing number of folders in the RCV process. no SMOTE = no oversampling correction of class imbalance is done.

nfolds	SMOTE			
	SVM	MLP2	MLP1	NB
10	9.85 ±0.63	7.54 ±0.16	10.23 ±0.8	9.83 ±0.3
5	9.67 ±0.49	7.65 ±0.19	9.67 ±0.26	9.83 ±0.53
4	9.6 ±0.17	7.65 ±0.02	9.71 ±0.23	9.83 ±0.13
3	9.73 ±0.18	7.57 ±0.06	9.69 ±0.07	9.98 ±0.17

	no SMOTE			
	SVM	MLP2	MLP1	NB

10	1.86 ±0.00	9.70 ±1.45	NA	11.23 ±1.37
5	2.04 ±0.00	9.16 ±0.82	NA	11.20 ±1.14
4	1.95 ±0.40	9.62 ±0.75	NA	11.29 ±1.32
3	2.22 ±0.45	9.60 ±0.52	NA	11.08 ±1.23

**Table 6:** Average ± standard deviation AUC performance of SVM, MLP1, MLP2, and NB for decreasing number of folders in the RCV process. no SMOTE = no oversampling correction of class imbalance is done.

nfolds	SMOTE			
	SVM	MLP2	MLP1	NB
10	0.597 ±0.022	0.539 ±0.022	0.643 ±0.020	0.654 ±0.014
5	0.587 ±0.010	0.55 ±0.018	0.634 ±0.011	0.653 ±0.014
4	0.585 ±0.008	0.548 ±0.021	0.63 ±0.009	0.655 ±0.008
3	0.584 ±0.009	0.55 ±0.011	0.628 ±0.010	0.653 ±0.011

	no SMOTE			
	SVM	MLP2	MLP1	NB

10	0.495 ±0.020	0.631 ±0.026	0.661 ±0.021	0.656 ±0.014
5	0.481 ±0.019	0.615 ±0.008	0.661 ±0.008	0.658 ±0.007
4	0.473 ±0.004	0.631 ±0.011	0.661 ±0.012	0.659 ±0.008
3	0.471 ±0.007	0.627 ±0.015	0.657 ±0.002	0.658 ±0.009

## Data Availability

Data will remain proprietary of the hospital until aggregation in a nationwide dataset.
